# Current understanding and distinct features of multifocal and multicentric breast cancers

**DOI:** 10.1002/cnr2.1851

**Published:** 2023-06-22

**Authors:** Elizabeth Avera, Lilia Valentic, Loan Bui

**Affiliations:** ^1^ Department of Biology University of Dayton Dayton Ohio USA

**Keywords:** biomarkers, breast cancer, heterogeneity, multicentric and multifocal

## Abstract

**Background:**

Multifocal (MF) and multicentric (MC) breast cancers are referred to as synchronous, multiple ipsilateral breast cancers; however, the definitions vary among the literature, which has made understanding and analyzing these diseases challenging.

**Recent findings:**

The incidence ranges from 1% to 60%, with a higher prevalence in pre‐menopausal women. MF and MC breast cancers, compared with unifocal breast cancers, tend to be more aggressive and are associated with lower survival rates, higher recurrence, and lymph node metastasis. Typically, patients with MF/MC breast cancers are treated with radical surgery, while breast conservation therapy may also be considered. Investigations have focused on elucidating the distinct biological features of MF/MC breast cancers, including the clonality of the cancers, the genetic alterations, and the impact of these features on disease aggressiveness and patient prognosis.

**Conclusion:**

These findings will broaden the understanding of these breast cancer subtypes and aid in the development of more tailored treatment plans for patients.

## 1 INTRODUCTION

Breast cancer can be categorized into different subtypes based on the number of carcinoma foci present within the same breast tissue. Unifocal (UF) breast cancer is defined by a singular breast carcinoma, while multifocal (MF) and multicentric (MC) breast cancers are referred to as multiple synchronous ipsilateral breast carcinomas. However, there is substantial variation in the definitions of MF and MC subtypes in the literature, most notably in the use of breast quadrants, the distance between foci, and the inclusion of in situ lesions. These varying definitions of MF/MC cancers can be attributed to the wide range of incidence, from 1% to 60%.^1^, ^2^, ^3^, ^4^, ^5^, ^6^, ^7^, ^8^, ^9^ The incidence rate of MF/MC breast cancers has increased in recent years partly due to the improvement of imaging techniques.^3^, ^10^ While it is still somewhat debated, MF/MC breast cancers, compared with UF breast cancers, tend to be more aggressive and have worse prognosis, including lower survival rates, higher recurrence rates, and increased lymph node metastasis.^1^, ^2^, ^4^, ^5^, ^6^, ^7^, ^9^, ^11^, ^12^, ^13^, ^14^ MF/MC breast cancers are more common in pre‐menopausal women than UF breast cancers.^2^, ^3^ Typically, MF/MC breast cancers are treated with radical surgical options; however, recent studies have explored the viability of breast conservation therapy (BCT) as a treatment option for patients with these breast cancer subtypes.^10^, ^15^, ^16^, ^17^, ^18^, ^19^ In addition, recent investigations have focused on understanding the distinct biological differences between MF/MC and UF breast cancers that could elucidate the underlying causes of the increased aggressiveness and poorer prognosis associated with MF/MC cancers to aid in the selection of better treatment plans for these patients. Some studies have focused on studying the clonality of multiple MF/MC lesions via the homogeneity and heterogeneity of their grades, histological types, molecular subtypes, and genetic profiles and the impact of these features on the tumors' aggressiveness and patient prognosis.^20^, ^21^, ^22^, ^23^, ^24^, ^25^, ^26^, ^27^, ^28^ Other studies have investigated the effects of MF/MC subtypes on the expression of molecular biomarkers, genes of canonical cancer pathways, and cancer stem cell biomarkers.^8^, ^25^, ^27^, ^29^, ^30^, ^31^, ^32^, ^33^, ^34^ In this paper, we aim to review and update the current understanding of MF/MC breast cancers in terms of definitions, incidence, prognosis, and treatment options available for these diseases. Furthermore, we highlight findings on biological features, namely the clonality and the distinct biomarkers found in MF/MC breast cancers. We only select original research publications (most are retrospective studies) and one systematic review in English. Google Scholar and PubMed are the two main electronic databases for searching relevant articles. The following keywords are used to narrow the aspects of MF/MC breast cancers: “multifocal and multicentric,” “breast cancer,” “MMBC,” “multiple tumors,” “biomarkers,” “prognosis,” and “treatment.” The selected studies are found to be published between 1995 and 2022 and conduct experiments with a sample size of at least 11 patients.

## 2 DISCREPANCIES IN THE DEFINITION OF MULTIFOCAL AND MULTICENTRIC BREAST CANCERS

The definitions of MF and MC breast cancers are inconsistent in the literature, especially regarding the location of foci in breast quadrants, the distance between foci, and the invasiveness of the foci (Table 1). Most authors accept that MF and MC breast cancers involve multiple distinct tumors in the same and different breast quadrants, respectively.^3^, ^5^, ^6^, ^7^, ^12^, ^21^, ^28^ Some studies combine MF and MC cases (typically referred to as MMBC) and define the diseases as two or more invasive carcinomas that are separated by benign tissue, regardless of whether they are in the same or different breast quadrants.^1^, ^4^, ^9^, ^11^, ^20^, ^35^ Some authors additionally consider the distance between tumors in their definitions. For example, MF breast cancer was defined as two or more lesions in the same breast quadrant or separated by less than 4 cm, while MC breast cancer was defined as lesions separated by more than 4 cm or in different breast quadrants.^36^ However, a wide range of distances between tumors has been reported, ranging from 1 to 5 cm.^8^, ^35^, ^37^ Furthermore, most MF/MC cancers are defined as exclusively invasive carcinomas, including ductal and/or lobular breast cancers; however, some studies also included in situ lesions.^5^, ^7^, ^8^, ^12^, ^16^, ^20^, ^28^, ^35^, ^37^, ^38^ In addition to the non‐standardized criteria for MF/MC breast cancers, conventional mammogram and gross pathological examination seem to be unable to detect all MF/MC cancers.^10^, ^36^ Therefore, if two or more tumors cannot be distinguished, it can lead to the classification of a UF tumor rather than multiple tumors.^11^ This lack of consensus on the definitions of MF and MC breast cancers has challenged the treatment and management of MF/MC cancers^16^; moreover, it makes studying and comparing the biological behavior of these diseases difficult.

**TABLE 1:** Discrepancies in definition criteria of multifocal and multicentric breast cancers.

Author	Year	Breast quadrants^a^	Distance (cm)	Ductal or lobular carcinoma	Includes in situ lesions
Dawson et al.^20^	1995	No	1 to >2.5	Both	Yes
Katz et al.^36^	2001	Yes	4	Both	No
Andea et al.^12^	2004	Yes	N/A	Both	Yes
Sardanelli et al.^38^	2004	Yes	N/A	Both	Yes
Coombs et al.^7^	2005	Yes	N/A	Both	Yes
Joergensen et al.^1^	2008	No	N/A	Both	No
Gentilini et al.^16^	2009	Yes	N/A	Both	Yes
Garimella et al.^21^	2009	Yes	N/A	Ductal	No
Wolters et al.^3^	2013	Yes	N/A	Ductal	No
Zhou et al.^4^	2013	No	N/A	Both	No
Neri et al.^6^	2015	Yes	N/A	Both	No
Kanumuri et al.^5^	2015	Yes	N/A	Both	Yes
Lang et al.^9^	2017	No	N/A	Ductal	No
Ilić et al.^8^	2017	No	5	Lobular	Yes
Fushimi et al.^35^	2019	No	1	Both	Yes
Ghamrini et al.^37^	2020	Yes	2 to 5	Both	Yes
Zehni et al.^11^	2021	No	N/A	Not specified	Typically invasive
Jeon et al.^28^	2022	Yes	N/A	Ductal	Yes

^a^ These studies consider breast quadrants (same for MF, different for MC). N/A: Not available.

## 3 VARYING INCIDENCE RATES AND POTENTIAL POOR PROGNOSIS OF MULTIFOCAL/MULTICENTRIC BREAST CANCERS

Many studies determined that roughly 10%–20% of breast cancer cases were MF or MC, although a wide range of MF/MC incidence was found, from 1% to 60%, partially because of the discrepancies in the definitions of these cancers.^1^, ^2^, ^3^, ^4^, ^5^, ^6^, ^7^, ^8^, ^9^, ^31^ Many cases of MF/MC breast cancer occurred within the age range of 35 to 64 years.^16^, ^39^ A higher incidence rate of MF/MC breast cancers was found in pre‐menopausal women than UF breast cancer.^2^, ^3^ The incidence rate of MF/MC cancers has increased gradually in recent years due to improvements in imaging and diagnostic techniques.^3^, ^10^ Conventionally, MF/MC cancers were identified via mammogram and gross pathological examination.^36^ The use of magnetic resonance imaging increased the sensitivity of the detection of invasive foci, especially in dense breast tissue, despite the persistence of high levels of false positives.^38^

MF/MC breast cancers are more aggressive than UF cancers. They tend to have worse prognosis, including lower survival rate, increased rate of recurrence, and increased lymph node metastasis.^1^, ^2^, ^3^, ^4^, ^5^, ^6^, ^7^, ^9^, ^11^, ^12^, ^13^, ^14^ Several studies determined that MC cases were even more aggressive than MF cases, with lower overall survival and more advanced TNM staging.^3^, ^5^ However, a number of studies reported inconclusive results regarding MMBC having worse prognosis than UF breast cancer.^2^, ^7^, ^12^, ^35^, ^40^ Some studies conducted multivariate analysis, whereas others focused on fewer factors. Furthermore, several authors suggested alternative methods to reevaluate MF/MC breast cancers regarding prognosis. Lynch et al. used conventional staging guidelines to determine the T stage of MF/MC breast cancers from the diameter of the largest identified lesion and found that MC breast cancers were associated with 5‐year recurrence‐free survival; however, multivariate analysis revealed that MF and MC subtype had no independent impact on prognosis.^2^ Similarly, based on tumor size and axillary lymph node status, another study determined that MF/MC cancers were associated with lower 5‐ and 10‐year survival rates than UF tumors; however, multivariate analysis did not support multifocality as a significant independent prognostic factor.^40^ Coombs et al. compared the two tumor staging methods and their association with axillary node metastasis.^7^ The conventional method clearly indicated that multifocality was a significant independent predictor of node positivity, while the method using aggregated diameters of all foci did not.^7^ In contrast, in a retrospective study, Fushimi et al. reported that MMBC, when evaluated using the conventional T staging method, might not be an indicator of worse prognosis in breast cancer. Instead, the sum of the invasive diameters of all lesions was a significant prognostic factor that impacted disease‐free survival.^35^ In another study, Andea et al. suggested using volume or surface area instead of diameter to evaluate tumor size. They reported a significantly higher frequency of axillary lymph node metastasis in the MF/MC group compared with its UF counterpart with similar volume or surface area.^12^

## 4 TREATMENT OPTIONS FOR MULTIFOCAL AND MULTICENTRIC BREAST CANCERS

Traditionally, mastectomy has been the preferred surgical treatment option for MF/MC breast cancers.^15^, ^16^, ^19^ BCT, an effective treatment option for UF breast cancer,^17^ has also been considered for treating MF/MC breast cancers.^10^, ^15^, ^16^, ^17^, ^18^ A study on 160 patients found that there was no significant difference between the percentages of successful implementations of BCT in UF breast cancer patients compared to MF/MC breast cancer patients.^18^ One study on 476 MF/MC patients found BCT with wide excisions to be an effective treatment, resulting in the 5 year cumulative incidence of only 5.1% local recurrence and 6.2% mortality.^16^ Furthermore, a similar study found 0% local and 5% distant recurrence in 36 patients with multiple synchronous breast cancers who received BCT as long as excisions included clear margins.^15^ However, another meta‐analysis using forest plot method to study 19 272 patients with either UF or MF/MC breast cancers found a higher local recurrence rate in MF/MC patients treated with BCT than UF patients treated with BCT, although there was no difference between MF/MC patients treated with BCT and mastectomy.^41^

In addition to surgery, MF/MC breast cancer patients typically receive adjuvant therapy. One study determined that MF/MC cancer patients were more likely to be treated with adjuvant chemotherapy than UF patients, while there was no difference in the number of patients who received adjuvant hormonal therapy compared to UF patients.^17^ However, another study found that MF/MC patients (87.6% ER+, 78.2% PR+, over‐expressed 13.3% HER2) who had undergone BCT were more likely to receive hormonal or both chemo‐ and hormonal therapy than chemotherapy alone.^16^ Many patients are also treated with radiotherapy either during or after their surgical treatment, especially if they have undergone BCT.^15^, ^16^, ^17^ Patients diagnosed with MF/MC breast cancer may also undergo upfront axillary dissection and/or sentinel lymph node dissection to check for lymph node metastasis.^16^

Many recent studies have aimed to identify distinct characteristics in multiple foci to suggest more tailored treatment options rather than one common treatment applied for all MF/MC breast cancer patients. Some studies focused on studying the homogeneity and heterogeneity of MF/MC lesions, while others evaluated the expression of specific biomarkers in these cancer lesions. Details of these studies are discussed in the following sections.

## 5 HETEROGENEITY AMONG MULTIPLE TUMORS IN MULTIFOCAL/MULTICENTRIC BREAST CANCER PATIENTS

It remains unclear whether the multiple foci of MF/MC breast cancers develop simultaneously and independently or whether they originate from one primary tumor.^20^, ^22^, ^23^, ^25^, ^27^, ^28^, ^29^ A subsequent question then arises: whether the cancer cells from these foci are heterogeneous or homogeneous. Therefore, many studies have investigated the similarities and differences between these multiple, synchronous tumors in terms of tumor grades, histological and molecular subtypes. Because few studies report homogeneity, for ease of discussion, we have converted the homogeneity results into heterogeneity (Table 2). Regarding tumor grades, Garimella et al. identified that 16.7% of the foci in MF patients were heterogeneous, whereas Mosbah et al. found that only 3.3% of foci were heterogeneous in bifocal and bicentric breast cancer patients.^21^, ^24^ Regarding histological subtypes, a wide range of heterogeneity was found among foci: 7.9%–37.5%.^20^, ^22^, ^24^, ^26^, ^27^, ^42^ In addition, Dal et al. reported 15.7% heterogeneity within the largest foci.^27^ Regarding molecular subtypes, about 8.0%–10.1% of MF/MC patients exhibited heterogeneity across different foci.^22^, ^27^ Various research groups compared the expression of estrogen receptor (ER), progesterone receptor (PR), and human epidermal growth factor receptor 2 (HER2) between individual foci in MF/MC breast cancer patients. The heterogeneity of ER, PR, and HER2 ranged from 0.0% to 18.0%, 6.0% to 11.1%, and 0.0% to 6.0%, respectively.^21^, ^22^, ^23^, ^24^, ^28^

**TABLE 2:** Heterogeneity of multiple foci in multifocal and/or multicentric breast cancers.

Authors	Year	Size	Breast cancer description	Tumor grades histological subtypes	Molecular subtypes ER, PR, HER2
Dawson et al.^20^	1995	24	MF and MC	37.5% Histological types	N/A
Garimella et al.^21^	2009	18	MF	16.7% Tumor grades	0.0% ER^a^ 11.1% PR^a^
Choi et al.^22^	2012	65	MF and MC IDC	12% Tumor grades^a^ 37% Histological types^a^	8.0% Molecular subtypes 3.0% ER 11.0% PR 6.0% HER2
Pekmezci et al.^23^	2012	51	MF and MC	13.7% Tumor grades	7.8% ER and PR 2.0% HER2
Mosbah et al.^24^	2015	205	Bifocal and Bicentric	3.3% Tumor grades 10.3% Histological types	5.4% ER 7.3% PR 0.0% HER2
Boros et al.^42^	2015	155	MMBC	15.5% Histological types	N/A
Pawloski et al.^26^	2021	120	MF and MC	26.7% Histological types	N/A
Dal et al.^27^	2022	89	MF and MC	7.9% Histological types 15.7% Histological types within the largest foci	10.1% Molecular subtypes
Jeon et al.^28^	2022	17	MF	N/A	18.0% ER 6.0% PR 6.0% HER2

Abbreviations: IDC, Invasive ductal carcinoma; N/A, Not available.

^a^ Converted heterogeneity from the homogeneity results reported in the corresponding paper.

Several studies investigated the heterogeneity of axillary lymph node metastases and of the genetic profile of MF/MC breast cancers. Boros et al. determined that axillary lymph node metastases could also be heterogeneous (e.g., regarding histological type) from the foci in the breast, regardless of whether the foci were heterogeneous or homogeneous.^42^ Eeles et al. examined the clonality of MC breast cancers via *TP53* status, and found that foci were heterogeneous in 10%–25% of patients.^29^ Norton et al. analyzed the genetic profiles of 11 patients with MF/MC lobular breast cancers to compare the gene copy number and the gene expression in different foci and healthy tissues.^25^ The study revealed that different foci in each patient typically had similar gene copy numbers, with only 3 out of 80 genes (*CCND1*, *FADD*, *ORAOV1*) varying in two patients. However, 64% (466/730) of the genes had significantly different expression levels between foci; these included *CDH1* (E‐cadherin), *FIGF*, *RELN*, *SFRP1*, *MMP7*, *NTRK2*, *LAMB3*, *SPRY2*, and *KIT*.^25^

Although non‐heterogeneous tumors were prevalent, these studies highlighted the heterogeneity existing between MF/MC foci. Some authors agree that multiple foci mainly exhibit intramammary spread and are thus monoclonal; however, they may also arise independently.^20^, ^25^, ^29^ Most other studies do not make clear conclusions about the clonality or origins of the foci.^21^, ^23^, ^24^, ^26^, ^27^, ^28^, ^42^ In addition, changes that result in heterogeneity could occur during tumor progression and dissemination.^22^ The heterogeneity data presented in these studies could be beneficial for the prediction of metastasis and prognosis and the recommendation of different postoperative treatments.^22^, ^27^

## 6 GENETICS AND GENE EXPRESSION IN MULTIFOCAL/MULTICENTRIC TUMORS

Many studies further investigated distinct cancer biomarkers in MF/MC breast cancers.^8^, ^11^, ^27^, ^29^, ^30^, ^31^, ^32^, ^33^, ^34^ The main focuses include altered expression of tumor suppressors, cell adhesion proteins, and other features of canonical cancer pathways. Some studies also examined whether these biomarkers could serve as potential prognostic factors for MF/MC breast cancers.^11^, ^29^, ^31^, ^32^, ^33^ However, the findings are not always consistent across different studies. According to Eeles et al. in their study of *TP53* status and prognosis in MC breast cancers, overexpression of *TP53* could be important in predicting poor overall survival in MC breast cancer patients.^29^ In addition to *TP53*, *BRCA* genes are among the highest penetrance genes in breast cancer. McCroie et al. reported that *BRCA2* carriers had a significantly higher likelihood of developing MF/MC breast cancers than *BRCA1* carriers.^34^ Regarding the expression of other genes, Norton et al. studied 730 cancer‐related genes and identified 35 upregulated genes and 34 downregulated genes. Compared with non‐cancerous tissues, the expression of the collagen gene, *COL11A1*, and *PKMYT1* in MF/MC foci exhibited the highest fold change. Noting that loss of *CDH1* is the hallmark of lobular breast cancer, these authors also identified downregulated expression of *CDH1* in MF/MC cancers. There were nine other genes, *FIGF*, *RELN*, *PROM1*, *SFRP1*, *MMP7*, *NTRK2*, *LAMB3*, *SPRY2*, and *KIT* that had smaller fold change values than *CDH1*.^25^ Similarly, in another study, the E‐cadherin expression was found to be significantly lower in MF/MC breast cancers than in UF cancers.^30^ Furthermore, this group found no difference in the expression of MUC‐1, a lubricating transmembrane protein, and β‐catenin, a protein involved in both cell adhesion and gene transcription, between MF/MC and UF tumors.^30^ In a study conducted by Ilić et al., the authors examined P53, BRCA1, and E‐cadherin status in MF/MC lobular breast cancers and reported no significant correlation between MF/MC subtype and the expression of these three proteins.^8^ Finally, Demir et al. investigated the expression of ALDH‐1, a cancer stem cell biomarker, and found that ALDH‐1 could be an important predictor of poor prognosis in breast cancers; however, ALDH‐1 expression was not significantly associated with MF/MC cases.^31^

Several other studies examined gene expression of factors associated with molecular subtypes. Dal et al. found that approximately 75% of MF/MC tumors were ER+, PR+, and/or HER2−.^27^ The study by Ilić et al. on MF/MC lobular cancers reported a similar trend: 85.7% of tumors expressed ER, 77.8% expressed PR, and 9.3% expressed HER2. However, they did not find any significant differences in the expression of these markers in MF/MC lobular cancers compared with non‐MF/MC lobular breast cancers.^8^ In contrast, Zehni et al. reported lower expression of both ER (39%) and PR (30.4%) but found no significant differences in the expression of ER and PR between MF/MC and UF cases.^33^ In addition, they demonstrated that ER and PR expression did not affect the prognosis of MF/MC breast cancers but that expression was correlated with favorable prognosis in UF breast cancer patients.^33^ Furthermore, this research group investigated the involvement of other nuclear receptors, such as vitamin D receptor, retinoid X receptor, thyroid hormone receptor alpha 1 (THRα1), and thyroid hormone receptor alpha 2 (THRα2), in the prognosis of MF/MC breast cancer patients.^11^, ^33^ They found that vitamin D receptor and retinoid X receptor worsened prognosis in the MF/MC group but not the UF group.^11^, ^33^ While THRα2 expression was associated with improved disease‐free survival in MF/MC patients, THRα1 expression was not associated with a significant change in disease‐free survival.^11^ Another study investigated the expression of RIP140 and LCoR, which are nuclear receptor transcriptional coregulators correlated with ER signaling. The authors found that MMBC samples exhibited higher expression of both RIP140 and LCoR compared with UF samples. However, the inverse correlation with overall survival was shown only in the UF group.^32^

## 7 CONCLUSION

While some studies determined that MF/MC breast cancers had worse prognosis than UF breast cancers, specifically regarding increased rates of metastasis, lower survival rates, and higher rates of recurrence, other studies found that MF/MC subtype was not an independent prognostic factor in breast cancer. The lack of universal definitions and consistent descriptions of MF and MC breast cancers could contribute to the increasing discrepancies of the prognosis of these diseases found in the literature, along with the varying diagnostic and evaluation methods. Traditionally, patients diagnosed with MF/MC breast tumors undergo more radical treatment options, such as mastectomy; however, current research indicates that conservation therapy could also be an effective treatment option. Some researchers investigated the heterogeneity of MF/MC cancers and determined that some foci have varying degrees of heterogeneity regarding histological subtypes, tumor grades, molecular subtypes, and genetic profiles. However, determining the tumorigenesis and metastatic progression of these multiple foci remains challenging and requires further investigation. Finally, several studies found that MF/MC foci express distinct molecular and genetic markers that represent potential prognostic indicators and may indicate new therapeutic options for these patients.

## AUTHOR CONTRIBUTIONS

**Loan Bui:** Supervision (lead); visualization (lead); writing – original draft (equal); writing – review and editing (lead). **Elizabeth Avera:** Writing – original draft (equal); writing – review and editing (equal). **Lilia Valentic:** Writing – original draft (supporting); writing – review and editing (supporting).

## CONFLICT OF INTEREST STATEMENT

The authors declare no conflicts of interest.

## ETHICS STATEMENT

Not applicable.

## Data Availability

Data sharing is not applicable to this article as no new data were created or analyzed in this study.
